# Clinical application of ‘Sophia Observation withdrawal Symptoms‐Paediatric Delirium’ screening tool in Danish version: A feasibility study

**DOI:** 10.1111/scs.13073

**Published:** 2022-03-06

**Authors:** Rikke Louise Stenkjaer, Ingrid Egerod, Mala Moszkowicz, Gorm Greisen, Erwin Ista, Suzanne Forsyth Herling, Janne Weis

**Affiliations:** ^1^ Department of Neonatology Copenhagen University Hospital Rigshospitalet Copenhagen Denmark; ^2^ Department of Intensive Care Copenhagen University Hospital Rigshospitalet Copenhagen Denmark; ^3^ Department of Clinical Medicine University of Copenhagen Copenhagen Denmark; ^4^ Research Unit at Child and Adolescent Mental Health Center Capital Region of Denmark Copenhagen Denmark; ^5^ Department of Neonatology, Rigshospitalet University of Copenhagen Copenhagen Denmark; ^6^ Department of Pediatric Surgery Pediatric Intensive Care Erasmus MC – Sophia Children’s Hospital Rotterdam the Netherlands; ^7^ The Neuroscience Centre Copenhagen University Hospital Rigshospitalet Copenhagen Denmark

**Keywords:** children, critical care nursing, family‐centred care, feasibility studies, intensive care, newborn, paediatric delirium, paediatric intensive care unit, translation

## Abstract

**Aims and objectives:**

The aims of the present study were investigating the feasibility of: (1) using the Danish version of Sophia Observation withdrawal Symptoms‐Paediatric Delirium (SOS‐PD) screening tool in clinical practice and (2) comparing SOS‐PD performance to a child psychiatrist's assessment using the diagnostic criteria as a reference standard.

**Background:**

Critically ill children risk developing delirium potentially causing discomfort and suffering. Intensive care delirium has a fluctuating course complicating detection. Systematic screening during and after intensive care is central to manage paediatric delirium.

**Design and methods:**

We used a descriptive and comparative design. First aim: Bedside nurses were asked to evaluate their experience of using the SOS‐PD. Second aim: We compared the SOS‐PD performance with the child psychiatrist assessment in 50 children aged 4 weeks to 18 years.

**Results:**

Nurses found the Danish version of the SOS‐PD applicable and easy to use. Of the 50 children included, 13 were diagnosed with delirium by the child psychiatrist. Consistency was found between the SOS‐PD score and the child psychiatrist's assessment (88%). We found three false‐negative and three false‐positive SOS‐PD cases. The false‐negative cases could be explained by the differences in time periods for the assessments. SOS‐PD assessments covered the past 4 h, whereas the psychiatric assessments covered the past 24 h. We assume the false‐positive cases represent an acceptable inconsistency between the two assessment methods.

**Conclusions:**

The Danish version of the SOS‐PD appeared suitable for identifying paediatric delirium. Our results emphasised the importance of assessment at least once during each nursing shift to ensure delirium detection around the clock due to the fluctuating course of delirium.

**Relevance to clinical practice:**

Implementing the Danish SOS‐PD may increase awareness of this critical disorder by improving systematic identification of paediatric delirium in clinical practice paving the way for improved delirium prevention and management.

## INTRODUCTION

Critically ill children are at risk of developing paediatric delirium (PD), which may cause discomfort and suffering with symptoms such as disturbed sleep‐wakefulness, disorientation, inattention, hallucinations, anxiety, altered behaviour and mood swings [1]. PD is associated with increased ventilator days, longer hospital stays, increased mortality and costs [2, 3]. It is reasonable to assume that experiencing delirium during intensive care may contribute to the post‐intensive care syndrome and delusional memories in child survivors [4, 5]. Systematic PD screening during or after intensive care is needed to identify the disorder as well as monitoring the effectiveness of actions to prevent PD. The present study evaluates the feasibility of introducing a screening tool to identify PD in clinical practice.

## BACKGROUND

In the American psychiatry Diagnostic and Statistical Manual of Mental Disorders, Fifth Edition (DSM‐V), delirium is described as a neurocognitive disorder [1]. Delirium is manifested as an acute cerebral dysfunction with the following diagnostic criteria: disturbance in attention, awareness and cognition (memory deficit, disorientation, perception), development over a short period of time and tendency to fluctuate in severity during the course of a day [1]. Three types of delirium are described: hyperactive, hypoactive and mixed delirium [1]. The child with hyperactive delirium is characterised by turmoil, increased psychomotor activity, restlessness and combative behaviour while being insecure, anxious and vigilant [2, 6]. Conversely, the child with hypoactive delirium is remarkably quiet, withdrawn, sleepy and with a non‐demanding behaviour [2, 6]. The mixed type comprises alternating symptoms of hyper‐ and hypoactive type delirium [2, 6]. Diagnosis of delirium in adults focuses on cognitive changes or disruption, whereas emphasis is placed on behavioural changes in children [7, 8]. The incidence of PD in critically ill children reported in other studies varies from 5% to 57% depending on associated risk factors such as age <2 years, developmental delay, cardiac surgery and awareness of the condition [9, 10, 11, 12]. Many of the symptoms of PD overlap with other conditions often related to critical care such as pain, distress and iatrogenic withdrawal syndrome (IWS) [13]. Identifying and distinguishing between these conditions is important as they are treated differently. However, it can be challenging to distinguish these conditions without the use of validated screening tools.

At present, four tools for detection of PD have been validated in clinical practice: the paediatric Confusion Assessment Method for the ICU (p‐CAM‐ICU) [14]; the preschool Confusion Assessment Method for the ICU (ps‐CAM‐ICU) [6]; the Cornell Assessment of Paediatric Delirium (CAPD) [15] and Sophia Observation withdrawal Symptoms‐Paediatric Delirium screening tool (SOS‐PD) [16]. Both p‐CAM‐ICU and ps‐CAM‐ICU are assessment tools requiring patient interaction to assess delirium, as opposed to CAPD and SOS‐PD that are observational screening tools based on observations on behaviour during the past 4 h [6, 14, 15, 16]. In the present study, we evaluated the feasibility of the Danish version of the SOS‐PD using a child psychiatrist's assessment as a reference standard. We have chosen the SOS‐PD screening tool to ease application since it holds the advantage of providing concurrent assessment of PD and IWS, eliminating the need for using different tools for these two conditions [16, 17]. Furthermore, this screening tool is based on observations during the past 4 h taking the fluctuating nature of PD into account. To our knowledge, the SOS‐PD has not been evaluated in other countries than the Netherlands. Hence, in this study, we investigate the feasibility of the SOS‐PD application in clinical practice to identify barriers while preparing for a study validating the SOS‐PD.

The aims of the present study were investigating the feasibility of: (1) using the Danish version of Sophia Observation withdrawal Symptoms‐Paediatric Delirium (SOS‐PD) screening tool in clinical practice and 2) comparing the SOS‐PD performance with a child psychiatrist's assessment using DSM‐V as a reference standard.

## METHODS

### Design

In this feasibility study, we applied a descriptive and comparative design using qualitative and quantitative methods. We used the COnsensus‐based Standards for the selection of health Measurement INstruments (COSMIN) guideline [18], and international principles for translation and cultural adaption of patient reported outcome tools [19], including evaluation of the clinical application of the SOS‐PD.

### The SOS‐PD screening tool used by nurses

**FIGURE 1:**
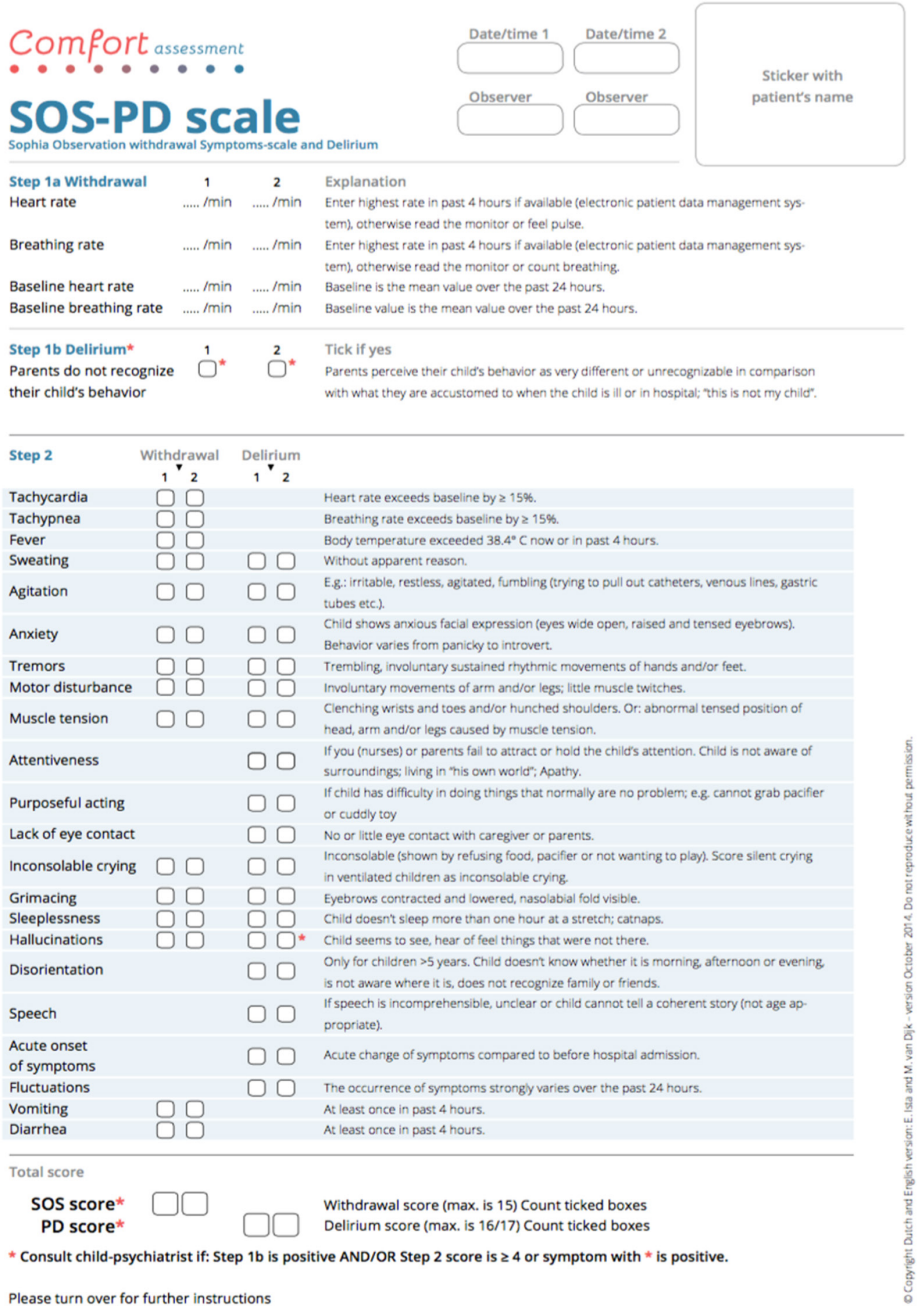
Sophia Observation withdrawal Symptoms‐scale – Paediatric Delirium (SOS‐PD). Published with permission from associate professor Erwin Ista

The SOS‐PD screening tool is an extended version of the 2009 Sophia observation withdrawal symptoms [20] validated at the Sophia Children's Hospital in the Netherlands to identify either PD or IWS, or both [16, 20]. The target population is children from 3 months to 18 years of age. SOS‐PD is an observational tool that does not require the cooperation of the child. Assessments are based on observations of the child's behaviour during the past 4 h. The tool includes 22 behavioural items whereof 10 items overlap in both conditions. The PD component consists of 17 items addressed to the nurses caring for the child, and one item addressed to the parents of the child (Figure 1). Items receive a score if the symptom has been present at any time during the observation period. The maximum score is 17 points. The cut‐off score indicating delirium is ≥4 [21]. The original Dutch SOS‐PD screening tool used by Paediatric Intensive Care Unit (PICU) nurses has shown a sensitivity of 92.3% and specificity of 96.5% when compared to a child psychiatrist's diagnosis of delirium [16].

The English version of SOS‐PD was translated into Danish according to principles of good practice for translation to ensure that the translation linguistically, conceptually and culturally was corresponding with the original tool [19]. Prior to translation, we obtained the permission from the original authors to translate the tool. Two authors of the present paper were certified to use the SOS‐PD by the leading member of the team that developed the tool. The training session consisted of three elements: (1) a theoretical introduction to PD, (2) an instruction on how to use the SOS‐PD screening tool and (3) PD assessment based on video recordings of three children. The video assessments were compared amongst the trainees and the instructor, and assessments were discussed with special focus on disagreements. Certifying the Danish authors ensured comprehension, correct use and interpretation of the SOS‐PD and approval of the process and the final translation [16].

### Assessment by child psychiatrist

The child psychiatrist performed a diagnostic assessment of the child using the Vanderbilt Assessment of Delirium in Infants and Children (VADIC) based on patient history from the medical record, interview with the child's parents and objective assessment of the child [8]. The VADIC is a valid bedside tool that has preserved the DSM‐V criteria with paediatric specified modifiers integrated for paediatric delirium screening [8]. VADIC compasses organising behavioural observations made at the moment and during the past 24 h within six domains: (1) level of consciousness, (2) mental status and perception, (3) attention and cognition, (4) sleep‐wake cycle, (5) affect and (6) language and thought, according to DSM‐V criteria [1]. The VADIC tool was used to ensure consistency and structure format for identifying PD [8].

### Setting

The evaluation of SOS‐PD was conducted at five different units at a University Hospital in Copenhagen treating children from 4 weeks to 18 years of age: the neonatal intensive care unit (NICU), paediatric semi‐intensive care unit, paediatric cardiac unit, paediatric cardiac intensive care unit and the general intensive care unit. All five units are the most specialised units in Denmark providing treatment and care for newborns and children with congenital malformations, heart diseases, trauma, respiratory distress and neurology.

### Sampling and data collection

Data were collected from May to December 2020 using 2–3 available patients on one assigned weekday each week during dayshift. Regarding the first aim of the study the participants evaluating the use of SOS‐PD were nurses caring for the included PICU patients on the day of the tool evaluation at the five hospital units. We conducted informal interviews with the nurses to determine their experience of the tool. Throughout the study, the PI used a logbook to record nurses’ comments and reflections on the usefulness of the tool, facilitators and barriers to the progress and recorded the immediate responses from the nurses when supervising the SOS‐PD assessment.

The second aim of the study was to compare the SOS‐PD performance used once a shift by the bedside nurse with the child psychiatrist assessment using VADIC. The children that were assessed were hospitalised 48 h or more, admitted to or transferred within the past week from an intensive care unit and lightly or non‐sedated (sedation level at COMFORT behaviour score >11 [22] or Richmond Agitation Sedation Scale > −3 [23]). We did not perform delirium assessment on deeply sedated children because the basic criteria for delirium will not be present. Training of nurses and key persons in the five units was promoted by close collaboration with the management teams (Table 1). The primary investigator (PI) identified children who met the inclusion criteria together with the unit key persons or the nurse leaders. Hereafter, the PI recruited 2–3 children and their parents to inform them of the study. All participating families received verbal and written information and gave written consent to participate. In a convenience sample, 50 patients were assessed. All assessments were performed by different bedside nurses and the child psychiatrist independently. If the bedside nurse had not completed the web‐based training program or received training from the key persons, the PI performed the assessment with the bedside nurse.

**TABLE 1:** Systematic training of nurses in PD and SOS‐PD

Elements in training program	Web based training program lasting 30 min – 2 modules	Other elements
1. Theoretical introduction to PD	1. Theoretical introduction to PD diagnosis, symptoms and risk factors	1. Key persons ensured focus and training in their own units, e.g. newsletters or posters
2. Introduction to Danish version of SOS‐PD	2. Introduction to Danish version of SOS‐PD with an integrated training session based on video recording of a child	2. Visibility of participating children was highlighted on whiteboards and action and reminder cards visibly placed
3. Discussion of PD assessments based on video recordings	3. Quiz with three questions about the subject in each module	
	4. Print out certificate	

Abbreviations: PD, Paediatric Delirium; SOS‐PD, Sophia Observation withdrawal Symptoms‐Paediatric Delirium.

### Data analysis

First study aim: Data on usability of SOS‐PD were bedside nurses’ statements that were recorded by the PI in the logbook. The qualitative data were analysed deductively focusing on elements for further face validation as well as facilitators and barriers to the application.

Second study aim: Data compared SOS‐PD and VADIC assessments. In this feasibility study, the data are not statistically significant, and we did not perform power calculations. The feasibility of the SOS‐PD assessment tool was calculated from standard definitions of true positive, true negative, false‐positive and false‐negative cases. In addition, the initial calculation of sensitivity and specificity, as well as positive and negative predicted values were included.

### Ethical considerations

The study was approved by the five management teams and the Danish Data Protection Agency (j.no.: P‐2019–548). According to Danish legislation approval from the National Committee on Heath Research Ethics for this type of study is not required (reference number: 19076015).

## RESULTS

### Feasibility of SOS‐PD in clinical practice

Based on comments and reflections noted by the PI in the logbook, all nurses generally found that the Danish SOS‐PD was quick and easy to use and had the advantage of concurrently assessing both PD and IWS. Furthermore, the nurses found it valuable to collaborate with the parents in the assessments, because the parents had a knowledge about the child's habitual mental condition before admission. Some of the items needed further clarification because the nurses were unsure of the items ‘tremors’ and ‘hunched shoulders’. These terms were not changed but the items were clarified in the supplementary text explaining the scoring of the items. In infants and young children <2 years of age, the nurses found challenges especially with the assessment of ‘speech’, ‘attentiveness’, ‘purposeful acting’ and ‘hallucinations’. The text explaining item scoring for ‘speech’ was clarified describing that language also included the non‐verbal communication and sounds, which the nurse or parent could imitate as the communication. The other terms were clarified and discussed in training sessions in how the terms could be seen in these young children.

### Feasibility of SOS‐PD performance

**FIGURE 2:**
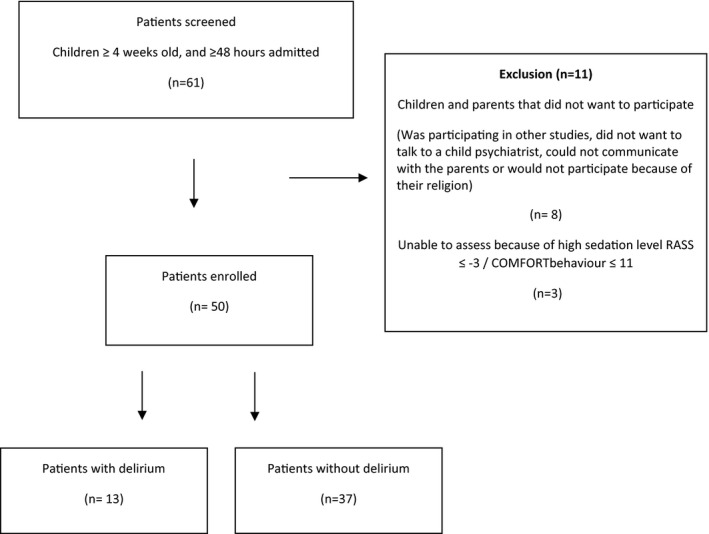
Flowchart of included children. Delirium according to reference. Psychiatrist assessments

The bedside nurses and the child psychiatrist assessed 50 children aged 4 weeks to 17 years. Figure 2 shows the flowchart of included children and Table 2 shows the children's characteristics. Nurses performed 69 SOS‐PD assessments in 50 children on the assigned weekdays. Many of the included children were ≤5 years of age (68%), had a cardiac diagnosis (54%) and did not require respiratory support at the assessment time (80%). Many of the SOS‐PD items were comparable to the areas observed by the child psychiatrist. However, the items ‘sweating’, ‘tremors’, ‘motor disturbance’, ‘muscle tension’ and ‘grimacing’ were not included in the psychiatric evaluation. The child psychiatrist focused on the child's baseline level of development before hospitalisation, patient history from the hospital chart, clinical behavioural observation made at the bedside and the parents’ description of the observations of their child's behaviour during the past 24 h. The key persons were familiar with the screening tool in clinical practice after completing the training program but implementing the use of the SOS‐PD around the clock was challenging. The nurses felt comfortable using the SOS‐PD, and they found the assessment essential in their clinical practice. Despite this, we found that the SOS‐PD was not used systematically once in every nursing shift as intended. The PI assisted the bedside nurse in 25% of the assessments in cases where the bedside nurse had not received training.

**TABLE 2:** Feasibility of SOS‐PD compared to DSM‐V criteria (*n* = 50)

	SOS‐PD	95% CI
Sensitivity	76.9%	46.2–95.0
Specificity	91.9%	78.1–98.3
Positive predictive value	76.9%	52.0–91.2
Negative predictive value	91.9%	80.7–96.9

Abbreviations: DSM‐V, American psychiatry Diagnostic and Statistical Manual of Mental Disorders, Fifth Edition; SOS‐PD, Sophia Observation withdrawal Symptoms‐Paediatric Delirium.

We calculated the consistency between the nurses’ and the psychiatrist's assessment and found agreement in 44 of 50 assessments (88%). The feasibility of SOS‐PD compared to DSM‐V criteria is shown in Table 2.

In all, there were six cases displaying inconsistencies between the SOS‐PD screening and the reference standard being the child psychiatrist's assessments. With a SOS‐PD cut‐off ≥4 there were three false‐negative PD assessments and three false‐positive (Table 3). The child psychiatrist identified 13 of the 50 assessed children as having a condition consistent with a PD disorder responding to a prevalence of 26%. The SOS‐PD identified 10 of these (Table 3). The inconsistencies in assessments leading to false‐negative results appeared mainly to be caused by the time scope of observation period. SOS‐PD scoring was based on observations during the past 4 h in the dayshift, whereas the psychiatric assessment included the past 24 h. In the three false‐negative cases, signs of delirium had been described in the patient records the previous evening and were also reported by the parents with children presenting signs of visual hallucinations. The SOS‐PD score, however, had not been performed in the evening, and the next day the score was <4. In all three false‐positive cases the SOS‐PD score was 4, just reaching the cut‐off score. We assume the false‐positive cases represent an acceptable inconsistency between the two different assessments performed by different people. Ten % of assessments were conducted in newborns and children up to 2 months of age (Table 4). In all these cases there was consistency between the SOS‐PD and the child psychiatrist's assessment.

**TABLE 3:** Number of assessments performed by nurses and psychiatrist

	Psychiatrist: delirium positive	Psychiatrist: delirium negative	Total
SOS‐PD ≥4: (delirium positive)	10	3	13
SOS‐PD <4: (delirium negative)	3	34	37
Total	13	37	50

Note

SOS‐PD ≥4 indicates delirium. SOS‐PD <4 indicates no delirium.

Abbreviation: SOS‐PD, Sophia Observation withdrawal Symptoms‐Paediatric Delirium.

**TABLE 4:** Descriptive characteristics of the study children with and without confirmed delirium by psychiatrist (*n* = 50)

Characteristics	All children (*n* = 50) Number (%)	Children with confirmed delirium (*n* = 13) Number (%)
Gender		
Female	13 (26)	1 (8)
Male	37 (74)	12 (92)
Age categories		
Newborn – 2 months	5 (10)	0 (0)
3–23 months	21 (42)	5 (38)
2–5 years	8 (16)	4 (31)
6–12 years	9 (18)	1 (8)
>12 years	7 (14)	3 (23)
Reason for admission		
Respiratory failure	6 (12)	0 (0)
Cardiac (including cardiac surgery)	27 (54)	7 (54)
Infections	3 (6)	1 (8)
Trauma/accident	4 (8)	2 (15)
Surgery (other than cardiac)	6 (12)	2 (15)
Transplantation	2 (4)	1 (8)
Neurology	2 (4)	0 (0)
Respiratory support at assessment		
None	40 (80)	11 (85)
Oxygen	3 (6)	2 (15)
Non‐invasive ventilation	1 (2)	0 (0)
Mechanical ventilation	6 (12)	0 (0)

The importance of parent participation in SOS‐PD screening emerged as an unexpected but important finding. Both nurses and the child psychiatrist became aware that parents’ contribution was critical for SOS‐PD and VADIC assessments. The parents were present most of the time with their child and were alert to any changes in attention, cognition and awareness.

## DISCUSSION

In this feasibility study, we evaluated the applicability of SOS‐PD in clinical practice and explored disagreements between the SOS‐PD and child psychiatrist's assessment as a reference standard in order to successfully adapt the Danish version for use in a Danish setting as well as identifying elements of importance for implementation of effective PD screening. Our results indicate that implementation of the Danish version of the SOS‐PD is feasible. SOS‐PD seemed easy to use in clinical practice with the adaptations made based on the nurses’ comments and experiences from the piloting patient cases. In general, the results of the SOS‐PD assessment were consistent with the child psychiatrist assessment with good sensitivity and specificity in spite of being a novel instrument to the nurses. More importantly, we did found inconsistency in 6 cases. It became clear from the false‐negative cases, that one SOS‐PD assessment during the dayshift is not enough to ensure detection of PD. Systematic assessment covering 24 h is needed due to the possible fluctuation of PD. This would also be in accordance with the timespan included by the child psychiatrist. SOS‐PD is intended to be applied three times a day [16], and it is actually recommended to assess for PD at least once per shift or as indicated by the child's condition [13]. In all three false‐positive cases, the SOS‐PD score just reached the cut‐off score at 4. A larger validation study is needed to judge if the cut‐off of 4 is optimal for the Danish version.

Focus on PD is a new area in Danish PICUs, but awareness of and interest in this condition is increasing. Nurses are familiar with assessment tools to identify pain and performing systematic assessments are part of nurses’ daily work in intensive care units in many countries [17, 24]. Recent studies, however, reveal that regular monitoring for PD was applied in less than one‐third of the participating PICUs [25, 26]. To remind the nurses to use the SOS‐PD during every shift, magnets and action‐ and reminder cards were placed in visible spaces, but even so the nurses did not use the SOS‐PD systematically around the clock as intended. All 50 children had at least one SOS‐PD assessment from dayshift probably because the PI prompted the bedside nurse on the assigned weekday to perform the SOS‐PD assessment. The nurses relied on their professional judgement and only used the tool if they suspected PD during evening and night shifts. Scepticism towards delirium screening tools is a common barrier along with knowledge deficits also described in other studies [27, 28, 29]. Suggestions for overcoming implementation barriers are described in several studies and includes: continuing education, flexible timing of assessment and computerised access to the screening tool [17, 28, 30, 31]. Nurse leaders play a key role to reduce barriers in practice with directing guiding, motivation and supporting staff during the implementation [32, 33]. In preparation for our future validation study, these barriers will be addressed by increased efforts by ensuring active participation of nurse managers to stimulate implementation, increased visual reminders, implementing a local guideline for PD assessment and integrating the SOS‐PD in the electronic patient record to ease access and documentation.

Understanding and implementing delirium monitoring and management for children require interdisciplinary contribution with collaboration amongst nurses, intensivists, paediatricians and psychiatrists. Only few physicians completed the web‐based program. In this study, the focus was mostly on the nurses because they had to become familiar with the tool to be able to identify PD. Critical care nurses work in close proximity to the child, observing and providing care throughout their shift, whereby they observe the child's suffering and discomfort. This may promote motivation to learn how to identify PD in order to subsequently alleviate this discomfort. PD assessment could advantageously be an integral part of interdisciplinary daily rounds, where the healthcare professionals systematically evaluate pain, anxiety, level of sedation and delirium in the context of the child's overall condition. To this end, The Paediatric Road Map is a systematic approach to promote the monitoring of pain, anxiety, level of consciousness and delirium [34].

In the present study, the items ‘purposeful acting’, ‘hallucinations’, ‘disorientation’ and ‘speech’ were difficult for the nurses to assess in infants less than 2 years of age. It has previously been established that assessment of infants and young children is challenging [6, 8]. The SOS‐PD screening tool is not validated for newborns and infants less than 3 months of age. Nevertheless, we found it reasonable to include the 4‐week old infants present in the neonatal unit because to our experience, these infants may display symptoms consistent with PD in older children. PD has previously been reported in NICU infants presenting classic symptoms of PD such as agitation, inconsolability, poor attention, restlessness and altered sleep‐wake cycle, where these infants were identified as having delirium by a psychiatrist as well as ICU staff using the CAPD assessment tool [35]. We included five infants below 2 months of age with complete agreement between the SOS‐PD and the child psychiatrist's assessment.

An unexpected finding was the importance of the parent's knowledge of their child for the nurses’ and child psychiatrist's assessments because they were alert to any changes in attention, cognition and awareness. This emphasises the necessity of shared responsibility and partnership between nurses and parents in accordance with key elements in the family centred care approach [36, 37]. It is described that parents want to be present and participate in the care of their child and that their child is calmer when they are involved [38, 39]. Parents also feel that their active participation may help them cope better with their own stress [38]. Studies show that delirium detection by the family in adult settings seems promising because family caregivers are better able to detect changes in patient cognition and behaviour than nurses, because they know their relatives from pre‐level and functioning [40, 41, 42]. This is most likely also true for parents of children with delirium. In a recent study it has been shown that children without a bedside parent was more likely to be screened positive for delirium [43]. Parental presence and involvement in PD detection and treatment should be explored in the future research.

### Limitations

One of the strengths in this feasibility study was the collaboration with the original developers of the SOS‐PD screening tool to ensure the translation process and the close collaboration with a child psychiatrist to ensure the accuracy of the translated version of SOS‐PD. A limitation was the relatively small number of children on mechanical ventilation. Most of these children were deeply sedated and were excluded as they did not fulfil inclusion criteria. Another limitation was that the nurses did not use SOS‐PD systematically around the clock.

## CONCLUSION AND RELEVANCE TO CLINICAL PRACTICE

This study substantiated the feasibility of using the Danish version of the SOS‐PD in critical care and intermediate paediatric care paving the way for systematic implementation in clinical practice. We successfully translated the screening tool into Danish and performed a preliminary evaluation of the SOS‐PD. The nurses found the tool applicable and easy to use and there was a high degree of agreement between the nurses’ assessments using SOS‐PD and the child psychiatrist's assessment. Our results indicate that the translated version of SOS‐PD is suitable for identifying PD and emphasise the importance of using the screening tool at least once during each nursing shift to ensure PD assessment around the clock as PD has a fluctuating course. Further studies are needed to validate the SOS‐PD and to improve prevention and treatment of PD through non‐pharmacological interventions including parental participation.

## CONFLICT OF INTEREST

None.

## AUTHOR CONTRIBUTIONS

**Rikke Louise Stenkjaer** conceived and designed the analysis, collected the data contributed data or analysis tools, performed the analysis and wrote the paper. **Ingrid Egerod** conceived and designed the analysis, contributed data or analysis tools, performed the analysis and wrote the paper. **Mala Moszkowicz** conceived and designed the analysis, collected the data and wrote the paper. **Gorm Greisen** conceived and designed the analysis, contributed data or analysis tools, performed the analysis and wrote the paper. **Erwin Ista** conceived and designed the analysis, contributed data or analysis tools and wrote the paper. **Suzanne Forsyth Herling** conceived and designed the analysis, contributed data or analysis tools, performed the analysis and wrote the paper. **Janne Weis** conceived and designed the analysis, contributed data or analysis tools, performed the analysis and wrote the paper.

## References

[1] American Psychiatric Association A . Diagnostic and statistical manual of mental disorders, 5th ed. Arlington, VA: American Psychiatric Association; 2013.

[2] Traube C , Silver G , Gerber LM , Kaur S , Mauer EA , Kerson A , et al. Delirium and mortality in critically ill children: epidemiology and outcomes of pediatric delirium. Crit Care Med. 2017;45(5):891–8. 28288026 10.1097/CCM.0000000000002324 PMC5392157

[3] Traube C , Mauer EA , Gerber LM , Kaur S , Joyce C , Kerson A , et al. Cost associated with pediatric delirium in the ICU. Crit Care Med. 2016;44(12):e1175–e9. 27518377 10.1097/CCM.0000000000002004 PMC5592112

[4] Manning JC , Pinto NP , Rennick JE , Colville G , Curley MAQ . Conceptualizing post intensive care syndrome in children‐the PICS‐p framework. Pediatric Crit Care Med. 2018;19(4):298–300. 10.1097/PCC.0000000000001476 29406379

[5] Colville G , Kerry S , Pierce C . Children's factual and delusional memories of intensive care. Am J Respir Crit Care Med. 2008;177(9):976–82. 18244955 10.1164/rccm.200706-857OC

[6] Smith HA , Gangopadhyay M , Goben CM , Jacobowski NL , Chestnut MH , Savage S , et al. The preschool confusion assessment method for the ICU: valid and reliable delirium monitoring for critically ill infants and children. Crit Care Med. 2016;44(3):592–600. 26565631 10.1097/CCM.0000000000001428 PMC4764386

[7] Silver G , Kearney J , Traube C , Atkinson TM , Wyka KE , Walkup J . Pediatric delirium: evaluating the gold standard. Palliat Support Care. 2015;13(3):513–6. 24762563 10.1017/S1478951514000212 PMC4968931

[8] Gangopadhyay M , Smith H , Pao M , Silver G , Deepmala D , De Souza C , et al. Development of the vanderbilt assessment for delirium in infants and children to standardize pediatric delirium assessment by psychiatrists. Psychosomatics. 2017;58(4):355–63. 28506544 10.1016/j.psym.2017.03.006 PMC5482775

[9] Traube C , Silver G , Reeder RW , Doyle H , Hegel E , Wolfe HA , et al. Delirium in critically Ill children: an international point prevalence study. Crit Care Med. 2017;45(4):584–90. 28079605 10.1097/CCM.0000000000002250 PMC5350030

[10] Patel AK , Biagas KV , Clarke EC , Gerber LM , Mauer E , Silver G , et al. Delirium in children after cardiac bypass surgery. Pediatr Crit Care Med. 2017;18(2):165–71. 27977539 10.1097/PCC.0000000000001032 PMC5658045

[11] Alvarez RV , Palmer C , Czaja AS , Peyton C , Silver G , Traube C , et al. Delirium is a common and early finding in patients in the pediatric cardiac intensive care unit. J Pediatr. 2018;195:206–12. 29395177 10.1016/j.jpeds.2017.11.064

[12] Schieveld JN , Leroy PL , van Os J , Nicolai J , Vos GD , Leentjens AF . Pediatric delirium in critical illness: phenomenology, clinical correlates and treatment response in 40 cases in the pediatric intensive care unit. Intensive Care Med. 2007;33(6):1033–40. 17457571 10.1007/s00134-007-0637-8 PMC1915613

[13] Harris J , Ramelet AS , van Dijk M , Pokorna P , Wielenga J , Tume L , et al. Clinical recommendations for pain, sedation, withdrawal and delirium assessment in critically ill infants and children: an ESPNIC position statement for healthcare professionals. Intensive Care Med. 2016;42(6):972–86. 27084344 10.1007/s00134-016-4344-1 PMC4846705

[14] Smith HA , Boyd J , Fuchs DC , Melvin K , Berry P , Shintani A , et al. Diagnosing delirium in critically ill children: Validity and reliability of the Pediatric Confusion Assessment Method for the Intensive Care Unit. Crit Care Med. 2011;39(1):150–7. 20959783 10.1097/CCM.0b013e3181feb489 PMC3776416

[15] Traube C , Silver G , Kearney J , Patel A , Atkinson TM , Yoon MJ , et al. Cornell Assessment of Pediatric Delirium: a valid, rapid, observational tool for screening delirium in the PICU. Crit Care Med. 2014;42(3):656–63. 24145848 10.1097/CCM.0b013e3182a66b76 PMC5527829

[16] Ista E , van Beusekom B , van Rosmalen J , Kneyber MCJ , Lemson J , Brouwers A , et al. Validation of the SOS‐PD scale for assessment of pediatric delirium: a multicenter study. Crit Care (London, England). 2018;22(1):309. 10.1186/s13054-018-2238-z PMC6247513 30458826

[17] Stenkjaer RL , Pedersen PU , Hundrup YA , Weis J . Evaluation of NICU nurses’ competence in pain assessment 5 years after implementation of the COMFORTneo Scale. Adv Neonat Care. 2019;19(5):409–15. 10.1097/ANC.0000000000000636 31517644

[18] Mokkink LB . COSMIN Study Design Checklist for Patient‐reported outcome measurement instruments; 2019.

[19] Wild D , Grove A , Martin M , Eremenco S , McElroy S , Verjee‐Lorenz A , et al. Principles of good practice for the translation and cultural adaptation process for patient‐reported outcomes (PRO) measures: report of the ispor task force for translation and cultural adaptation. Value Health. 2005;8(2):94–104. 15804318 10.1111/j.1524-4733.2005.04054.x

[20] Ista E , van Dijk M , de Hoog M , Tibboel D , Duivenvoorden HJ . Construction of the sophia observation withdrawal symptoms‐scale (SOS) for critically ill children. Intensive Care Med. 2009;35(6):1075–81. 19367394 10.1007/s00134-009-1487-3

[21] Ista E , Te Beest H , van Rosmalen J , de Hoog M , Tibboel D , van Beusekom B , et al. Sophia Observation withdrawal Symptoms‐Paediatric Delirium scale: a tool for early screening of delirium in the PICU. Aust Crit Care. 2018;31(5):266–73. 28843537 10.1016/j.aucc.2017.07.006

[22] Ambuel B , Hamlett KW , Marx CM , Blumer JL . Assessing distress in pediatric intensive care environments: the COMFORT scale. J Pediatr Psychol. 1992;17(1):95–109. 1545324 10.1093/jpepsy/17.1.95

[23] Sessler CN , Gosnell MS , Grap MJ , Brophy GM , O'Neal PV , Keane KA , et al. The Richmond Agitation‐Sedation Scale: validity and reliability in adult intensive care unit patients. Am J Respir Crit Care Med. 2002;166(10):1338–44. 12421743 10.1164/rccm.2107138

[24] Boerlage AA , Ista E , Duivenvoorden HJ , de Wildt SN , Tibboel D , van Dijk M . The COMFORT behaviour scale detects clinically meaningful effects of analgesic and sedative treatment. Eur J Pain (London, England). 2015;19(4):473–9. 10.1002/ejp.569 25070754

[25] Kudchadkar SR , Yaster M , Punjabi NM . Sedation, sleep promotion, and delirium screening practices in the care of mechanically ventilated children: a wake‐up call for the pediatric critical care community*. Crit Care Med. 2014;42(7):1592–600. 24717461 10.1097/CCM.0000000000000326 PMC4061156

[26] Staveski SL , Pickler RH , Lin L , Shaw RJ , Meinzen‐Derr J , Redington A , et al. Management of pediatric delirium in pediatric cardiac intensive care patients: an international survey of current practices. Pediatr Crit Care Med. 2018;19(6):538–43. 29863637 10.1097/PCC.0000000000001558

[27] Trogrlic Z , Ista E , Ponssen HH , Schoonderbeek JF , Schreiner F , Verbrugge SJ , et al. Attitudes, knowledge and practices concerning delirium: a survey among intensive care unit professionals. Nurs Crit Care. 2017;22(3):133–40. 26996876 10.1111/nicc.12239

[28] Flaigle MC , Ascenzi J , Kudchadkar SR . Identifying Barriers to Delirium Screening and Prevention in the Pediatric ICU: evaluation of PICU Staff Knowledge. J Pediatr Nurs. 2016;31(1):81–4. 26362671 10.1016/j.pedn.2015.07.009 PMC4724532

[29] Norman SL , Taha AA . Delirium knowledge, self‐confidence, and attitude in pediatric intensive care nurses. J Pediatr Nurs. 2019;46:6–11. 30802805 10.1016/j.pedn.2019.01.013

[30] Franken A , Sebbens D , Mensik J . Pediatric delirium: early identification of barriers to optimize success of screening and prevention. J Pediatr Health Care. 2019;33(3):228–33. 30449648 10.1016/j.pedhc.2018.08.004

[31] Rohlik GM , Fryer KR , Tripathi S , Duncan JM , Coon HL , Padhya DR , et al. Overcoming barriers to delirium screening in the pediatric intensive care unit. Crit Care Nurse. 2018;38(4):57–67. 30068721 10.4037/ccn2018227

[32] Weis J , Zoffmann V , Egerod I . Improved nurse‐parent communication in neonatal intensive care unit: evaluation and adjustment of an implementation strategy. J Clin Nurs. 2014;23(23–24):3478–89. 24698260 10.1111/jocn.12599

[33] Salmela S , Eriksson K , Fagerström L . Leading change: a three‐dimensional model of nurse leaders’ main tasks and roles during a change process. J Adv Nurs. 2012;68(2):423–33. 21848926 10.1111/j.1365-2648.2011.05802.x

[34] Smith HA , Brink E , Fuchs DC , Ely EW , Pandharipande PP . Pediatric delirium: monitoring and management in the pediatric intensive care unit. Pediatr Clin North Am. 2013;60(3):741–60. 23639666 10.1016/j.pcl.2013.02.010

[35] Groves A , Traube C , Silver G . Detection and management of delirium in the neonatal unit: a case series. Pediatrics. 2016;137(3):e20153369. 26908706 10.1542/peds.2015-3369

[36] Mikkelsen G , Frederiksen K . Family‐centred care of children in hospital ‐ a concept analysis. J Adv Nurs. 2011;67(5):1152–62. 21272055 10.1111/j.1365-2648.2010.05574.x

[37] Brødsgaard A , Pedersen JT , Larsen P , Weis J . Parents’ and nurses’ experiences of partnership in neonatal intensive care units: a qualitative review and meta‐synthesis. J Clin Nurs. 2019;28(17–18):3117–39. 31112337 10.1111/jocn.14920

[38] Axelin A , Salanterä S , Lehtonen L . ‘Facilitated tucking by parents’ in pain management of preterm infants‐a randomized crossover trial. Early Human Dev. 2006;82(4):241–7. 10.1016/j.earlhumdev.2005.09.012 16410042

[39] Ames KE , Rennick JE , Baillargeon S . A qualitative interpretive study exploring parents’ perception of the parental role in the paediatric intensive care unit. Intensive Crit Care Nurs. 2011;27(3):143–50. 21511474 10.1016/j.iccn.2011.03.004

[40] Krewulak KD , Sept BG , Stelfox HT , Ely EW , Davidson JE , Ismail Z , et al. Feasibility and acceptability of family administration of delirium detection tools in the intensive care unit: a patient‐oriented pilot study. CMAJ Open. 2019;7(2):E294–e9. 10.9778/cmajo.20180123 PMC6488481 31028053

[41] Steis MR , Evans L , Hirschman KB , Hanlon A , Fick DM , Flanagan N , et al. Screening for delirium using family caregivers: convergent validity of the Family Confusion Assessment Method and interviewer‐rated Confusion Assessment Method. J Am Geriatr Soc. 2012;60(11):2121–6. 23039310 10.1111/j.1532-5415.2012.04200.x PMC3498543

[42] Bohart S , Merete Møller A , Forsyth HS . Do health care professionals worry about delirium? Relatives’ experience of delirium in the intensive care unit: a qualitative interview study. Intensive Crit Care Nurs. 2019;53:84–91. 31079979 10.1016/j.iccn.2019.04.010

[43] Staveski SL , Pickler RH , Khoury PR , Ollberding NJ , Donnellan AL , Mauney JA , et al. Prevalence of ICU delirium in postoperative pediatric cardiac surgery patients. Pediatr Crit Care Med. 2021;22(1):68–78. 33065733 10.1097/PCC.0000000000002591

